# Improvement Schemes for Bacteria in MICP: A Review

**DOI:** 10.3390/ma17225420

**Published:** 2024-11-06

**Authors:** Jin Zhu, Renjie Wei, Jie Peng, Di Dai

**Affiliations:** 1 School of Civil Engineering, Wanjiang University of Technology, Maanshan 243031, China; xiaojinziwin@163.com; 2 Key Laboratory of Ministry of Education for Geomechanics and Embankment Engineering, Hohai University, Nanjing 210098, China; daidiruiko@ycit.edu.cn; 3 College of Civil Engineering, Yancheng Institute of Technology, Yancheng 224051, China

**Keywords:** MICP, bacterial properties, influencing factor, improvement schemes

## Abstract

Biomineralization is a common phenomenon in nature, and the use of microbial-induced calcium carbonate precipitation (MICP) technology for engineering construction is a successful attempt to utilize natural biological phenomena, which has become a hot topic of current research. There are many factors affecting MICP, such as bacterial properties and external environmental factors. Many scholars have carried out a lot of research on these factors, but even under appropriate conditions, the MICP process still has the problem of low efficiency. According to different engineering, the tolerance and effect of bacteria in different environments are also different. At the same time, the cultivation and preservation of bacteria will also consume a large amount of raw materials, which is far more significant than the cost of engineering construction. The efficiency and cost limit the large-scale application of this technology in practical engineering. In response to these problems, researchers are exploring new ways to improve the efficiency of MICP technology. Based on the bacteria used in MICP, this paper explores the mechanism of bacteria in the process of MICP and reviews the improvement of bacteria from the perspective of efficiency improvement and economy.

## 1. Introduction

Biomineralization is a biologically mediated process of material formation, which is intrinsically linked to the metabolic activities of organisms and centered on the activities of living cells [1]. Among them, a series of biochemical reactions caused by microbial growth and metabolism induce the precipitation of carbonate, phosphate, and other precipitates, which is an important part of the formation of surface minerals. In recent years, the use of microbial-induced calcium carbonate precipitation (MICP) for engineering construction has been successfully attempted [2]. The principle is that the metabolites of specific bacteria synthesize calcium carbonate with substances in the surrounding environment [3,4,5,6,7]. When the reaction is completed, the remaining microorganisms die due to the lack of a nutrient supply, which has less impact on the environment. It has the advantages of easy control, convenient operation, green environmental protection, and good ecological compatibility. Among MICP microorganisms, urea hydrolysis microorganisms have better effects. Taking *Sporosarcina pasteurii* as an example, bacteria produce urease, which catalyzes urea hydrolysis to increase the pH value of the surrounding solution, thereby promoting the formation of ammonium ions and carbonate ions. In the presence of calcium ions, the precipitation reaction occurs to form calcium carbonate crystals. Calcium carbonate induced by microorganisms has excellent properties, which allow it to be widely used in different engineering fields.

In terms of engineering applications, the low viscosity of MICP cements allows for better penetration into porous materials for reaction. Compared to ordinary Portland cement, less binder is consumed to obtain the same compressive strength as MICP cementing [8]. During the MICP process, bacteria have limited tolerance to calcium ions in a single injection, but bacteria are highly survivable in natural environments and the desired cementation of the treated material can be achieved by multiple injections of cementing solution. Within 8–12 h, bacteria can consume the cementing solution from a single injection, and excellent cementing results can be achieved through 5–12 injections [9]. Compared to cement, it has a short treatment cycle and no CO_2_ emissions. Meanwhile, biological calcium carbonate can be used as a filling material to seal cracks and pores, and when compared with calcium carbonate generated by chemical reactions, calcium carbonate induced by microorganisms has excellent cementing properties. This enables MICP to have good applicability in applications such as soil reinforcement [10,11], wind erosion control [12,13], crack repair [14,15,16], seepage control [17], liquefaction resistance [18,19], and cultural relics restoration [20,21]. During the MICP reaction process, bacterial urease decomposes urea to raise the reaction pH and generate CO_3_^2−^, which can react with some heavy metal ions to generate precipitates, and, at the same time, bio-carbonate has adsorptive properties, which makes MICP suitable for heavy metal remediation and immobilization [22]. In addition, MICP also has great potential in some emerging fields, such as carbon sequestration [23] and 3D printing [24]. In all applications of MICP, calcium carbonate is the key factor through which the effect of treatment is achieved. The parameters of biological calcium carbonate affect the treatment results, such as crystal size, morphology, generation rate, and distribution uniformity. While calcium carbonate is mainly generated through a bacterial reaction, the bacterial concentration, urease activity, and interaction between bacteria and calcium carbonate will affect the final treatment result. Meanwhile, environmental factors (e.g., temperature, pH, and salt concentration, etc.) not only directly affect the crystal generation, but also affect the bio-calcium carbonate properties by influencing the bacteria, the urease enzyme, and, thus, the bio-calcium carbonate properties.

A large number of studies in the literature has investigated factors that influence MICP [25,26,27], and how to improve the efficiency of MICP processing to respond to unfavorable environments has become a large focus of research. These research results provide a good solution to improve the MICP reinforcement effect. Overall, research in MICP technology has made some progress. However, the technique still has many drawbacks, such as low reinforcement efficiency and the generation of ammonia as a by-product. In order to promote the application of MICP in practical engineering and, at the same time, to provide reference for the research work in the field of MICP, we analyzed and studied the urease-producing bacteria (mainly *Sporosarcina pasteurii*) from the perspective of their characteristics and urease activity, reviewing the schemes of bacteria improvement in MICP.

## 2. Mechanism of Urease Action and the Role of Bacteria in MICP

### 2.1. The Mechanism of MICP Reaction at the Molecular Level: The Effect and Source of Urease

**Figure 1:**
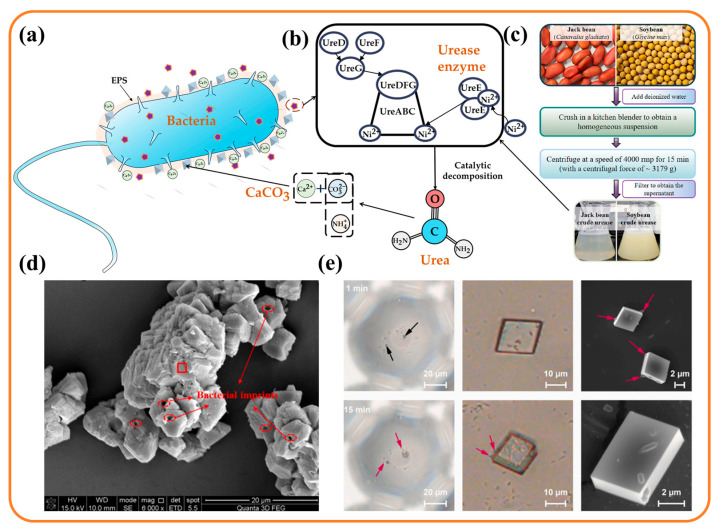
(**a**) Urease-producing bacteria; (**b**) urease gene; (**c**) extraction of urease from plants [36]; (**d**) bacteria as nucleation sites [43]; (**e**) bacteria–crystal interaction (The black arrows indicate the nucleation sites of CaCO_3_, and the red arrows indicate cells attached to the CaCO_3_ surface) [44].

During the MICP reaction, urease is the key protease that has the ability to induce mineralization. Numerous studies on urease gene composition and expression regulation have been conducted in the literature, and most urease-producing bacteria, including *Sporosarcina pasteurii*, contain seven and more genes such as ureA, B, C, D, E, F, and G. ureA, ureB, and ureC are the three subunit genes of urease, and ureD, ureE, ureF, and ureG are the auxiliary proteins necessary for urease activity genes [28], as shown in Figure 1b. It has been demonstrated that mutations in specific genes of ureD, ureF, and ureG dramatically disrupt the overall cellular urease activity [29]. ureE is the only nickel ion chaperone protein among the auxiliary proteins [30] and assists in the loading of nickel ions into the urease active site [31], and if some of the ureE genes are deleted, the urease activity of the bacteria is reduced by 50% [32]. Although urease activity has been found in numerous species of microorganisms, only in a few microorganisms, represented by *Sporosarcina pasteurii*, was extremely high urease activity found, inducing biomineralization reactions.

In addition to the direct use of bacterial solution, urease can also be extracted from bacteria and the induced mineralization (EICP) process can be carried out directly with urease solution (Figure 1c). The average size of *Sporosarcina pasteurii* cells is about 2800 nm [33], which is more than 200 times larger than the size of the urease molecule, and induced mineralization by the direct extraction of urease can solve the problem of the poor physical mobility of microorganisms in soils with small pore spaces [34]. The source of urease can be obtained not only by crushing bacterial cells, but also plant cells, with certain varieties of beans, watermelon seeds, and the pine family being rich in urease [35]. However, purified urease is expensive and may be uneconomical for large-scale applications, and some researchers have advocated the use of crude plant extracts as an economical alternative to purified urease, which includes crude extracts of jack beans [36], watermelon seeds [37], and soybeans [38,39]. The direct use of urease in EICP eliminates the process of bacterial culturing and bacterial metabolism to produce urease, avoids the influence of the external environment on bacterial division and growth, and does not cause a competition effect with other microorganisms. Moreover, there is no need to consume oxygen during the whole biochemical process, which can reinforce the deep soil [40]. It is worth noting that the activity of urease extracted from bacteria is usually two to three times that of the original bacterial solution, but its activity decays faster than that of the original bacteria [41,42].

### 2.2. The Role of Bacteria in the MICP Process

As well as producing urease, bacteria also influence calcium carbonate distribution and the crystal type and morphology [25]. The extracellular polymeric substance (EPS) matrix of proteins and polysaccharides on the bacterial cell surface controls calcium carbonate crystal type, morphology, and size, and the EPS maintains bacterial viability by acting as a protective template with various forms of slime or microencapsulated polysaccharides [45,46,47]. However, the composition and function of the EPS is characterized by a variety of properties, with heteropolysaccharides consisting of high-molecular-mass residues such as glucose, galactose, mannose, arabinose, and fructose, as well as negatively charged residues of carboxyl, phosphate, amine, and hydroxyl groups. The adhesive properties of bacterial EPSs are beginning to be recognized as providing nucleation sites during MICP [43,48], as shown in Figure 1d. Due to its adhesive and negatively charged characteristics, an EPS can incorporate nearby metal cations, such as Mg^2+^, Fe^3+^, Mn^2+^, and Ca^2+^, and induce the encrustation of microbially mediated extracellular calcium carbonate, thereby accelerating the aggregation of bacteria and calcium carbonate [49,50]. Oxygen concentration, pH, temperature, medium composition, and salt ion concentration affect the composition and amount of the EPS [49,51], resulting in the different morphology and mineralogy of those induced by bacterial species. In Figure 1e, the calcium carbonate crystals continue to accumulate and become larger as the MICP proceeded, at which time the calcium carbonate crystals had an adsorption effect on the bacteria [44], affecting the bacterial activity. The EPS–mineral–bacteria interactions still need to be further investigated.

### 2.3. Different Treatments of Bacteria in MICP: Bio-Stimulation and Bio-Augmentation

**Figure 2:**
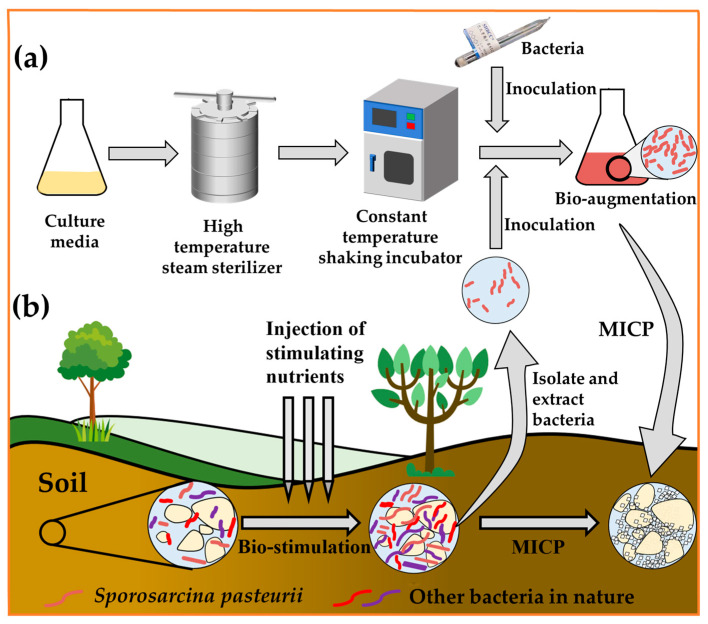
(**a**) Bio-augmentation; (**b**) bio-stimulation.

The effectiveness of MICP bio-cementation for soil improvement has been extensively demonstrated at the laboratory scale, and most of these studies have relied on bio-augmentation by injecting specific strains with specific metabolic capabilities, such as *Sporosarcina pasteurii*, to accomplish the biogeochemical process [52]. The method of bio-augmentation requires the bacteria to be cultivated in the laboratory and then carried out in the field, and different environmental factors in the field have a greater impact on the bacteria, such as temperature, pH, salt concentration, and the competition with in situ microbial communities. To overcome the drawbacks of bio-augmentation, the bio-stimulation approach was proposed for MICP-reinforced soil. The steps of the two different treatments are shown in Figure 2. Graddy et al. [53] conducted soil column experiments to assess microbial performance in the MICP for bio-augmentation and bio-stimulation, indicating that the approach using bio-stimulation was successful on all three replicate soil columns and that an earlier large-scale comparison between bio-stimulation and bio-augmentation suggested the equivalency of the two approaches. In terms of mechanical properties, including small-strain shear stiffness, strength, and cone penetration resistance, the improvement of bio-stimulation was generally comparable to that of bio-augmentation at similar cementation levels [54]. Cheng et al. [55] used the soil sample with the lowest bacterial enrichment activity to conduct in situ excitation tests on sand columns. The results showed that the method could be applied to both fine sand columns and coarse sand columns. After 10 cementing solution treatments, the unconfined compressive strength varied between 850~1560 kPa (coarse sand) and 150~700 kPa (fine sand). The strength and CaCO_3_ content of the bio-cemented sand column indicate that it is feasible to use in situ cultured native bacteria for bio-cementation.

However, for the treatment of bacteria, the choice is not binary. Based on MICP technology, Wang et al. [56] isolated a highly efficient strain named XR1# from soil, which has the advantage of simultaneously inducing mineralization and producing polysaccharides. In addition, the two metabolites can be artificially regulated to make the combination of calcium carbonate crystal filling and extracellular polysaccharide bonding, and the cemented sand column has the advantages of high yield, the uniform distribution of calcium carbonate, high strength, and good ductility. The method of isolation and extraction from the in situ environment followed by a bacterial culture is not only well adapted to the extraction environment, but can also enrich the culture to achieve the required bacterial concentration. The author evaluates different bacterial treatment methods in terms of environmental suitability, time cost, environmental dependence, and raw material consumption, as shown in Table 1. In addition to the above factors, the decision on which treatment option to use must also take into account the reaction transport conditions that control sedimentation localization, as well as the MICP treatment scope and calcium carbonate distribution.

However, the bio-stimulation approach may not always achieve the results of the bio-augmentation approach, especially when the initial concentration of native microorganisms is limited or the existing environmental conditions do not support the growth of native microbial species, and the bio-stimulation approach is more environmentally dependent. At the same time, the method of isolation and extraction from the in situ environment and then the bacterial culture consumes more time and raw materials. Currently, the dominant bacterial source of MICP is still through bio-augmentation; this paper focuses on a review of bacterial improvement based on bio-augmentation.

### 2.4. Assessment Indicators for Efficiency Improvements in MICP

**Figure 3:**
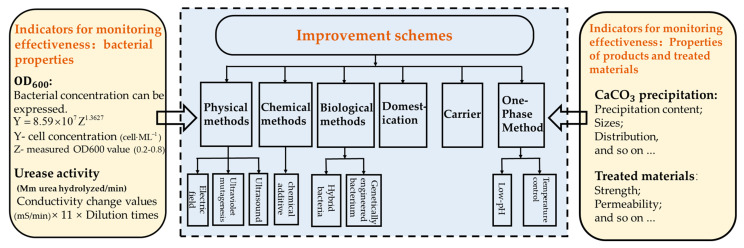
Main content and monitoring effectiveness indicators.

MICP efficiency can be improved by different processing methods. In this paper, by searching PubMed, Cochrane Central Register of Controlled Trials, Web of Science, EMBASE, and Scopus databases for studies involving microbial mineralization and improvement programs from the years 2000 to 2024, starting from the improvement of urease-producing bacteria in MICP, the effects of different improvement programs on MICP were summarized. The main content and structure are shown in Figure 3.

The effectiveness of the biomineralization technology can be monitored in terms of the properties of the bacteria themselves, the properties of the calcium carbonate produced, and the properties of the treated materials.

**Figure 4:**
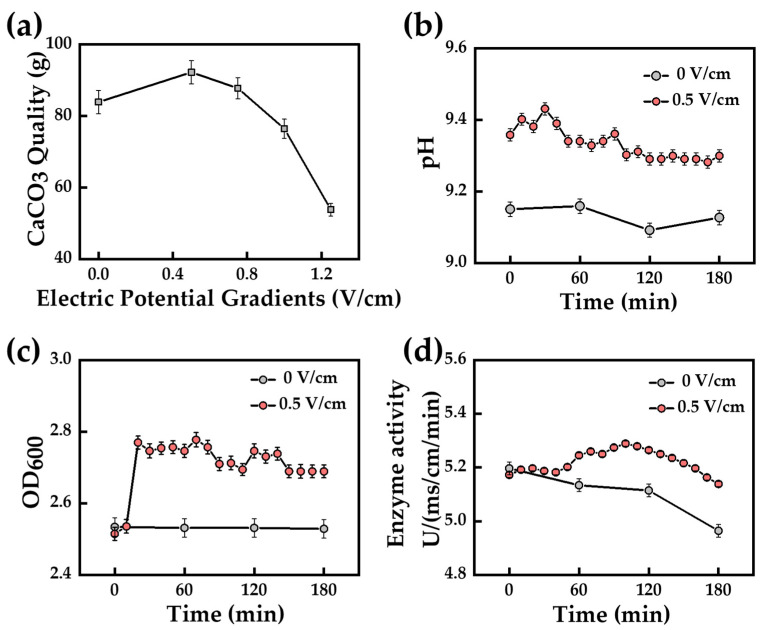
Effect of electric field on microbial performance: (**a**) Relationship between electric potential gradients and CaCO_3_ quality; (**b**) pH changes at different electric potential gradients; (**c**) OD_600_ values at different electric potential gradients; (**d**) Enzyme activity at different electric potential gradient (Adapted from Deng et al. [65]).

In the field of MICP, urease-producing bacterial indicators mainly include OD_600_ and urease activity. During the experiments, the biomass concentration was expressed as the optical density. Optical density was measured using a spectrophotometer at a wavelength of 600 nm [57]. The absorbance is positively correlated with the concentration of the substance in the bacterial solution; that is, the absorbance is positively correlated with the turbidity of the bacterial solution, so it is often used in microbiological tests as a method to determine the concentration of bacteria. In the literature reviewed in the text, OD_600_ was used to express the concentration of bacteria, and the calculation is demonstrated in Figure 4 [58]. Bacterial activity plays an important role in the rate of the hydrolysis of urea. The conductivity method was used to determine bacterial activity in the absence of calcium ions. The bacterial suspension to be tested was mixed 1:9 with 1 M urea solution. The change in the conductivity was measured three times continuously for 5 min using a conductivity meter, and the three results were averaged to calculate the change in the conductivity of the solution per minute. The result was converted to determine the rate of the urease hydrolysis of urea, which represents the urease activity [59].

The properties of the precipitated calcium carbonate and the properties of the treated materials are monitored differently depending on the treatment object. The properties of the precipitated calcium carbonate and the properties of the treated materials are monitored differently depending on the treatment object. The content of calcium carbonate can be tested by weighing [60] or by acid washing [61]. Calcium carbonate size is monitored with the use of instruments such as microscopes. In the case of soil reinforcement and concrete crack repair, the main focus is on testing the mechanical properties of the MICP-treated material, such as strength, pore properties, and permeability.

## 3. Physical, Chemical, and Biological Methods for Improving Bacterial Performance

### 3.1. Physical Method

#### 3.1.1. Electric Field

Electric fields affect the growth and metabolism of cells, and electric field stimulation can promote or kill microorganisms in two different ways. The implementation of electric field stimulation with small amplitude DC or AC electric fields will promote cell metabolism, gene expression, cell proliferation, enzyme activity, and cell membrane permeability and will even affect intracellular free radical reactions and the synthesis of biopolymers (such as DNA) [62]. In recent years, electric field stimulation technology has been widely used for its promotion in microbial engineering [63,64].

**Figure 5:**
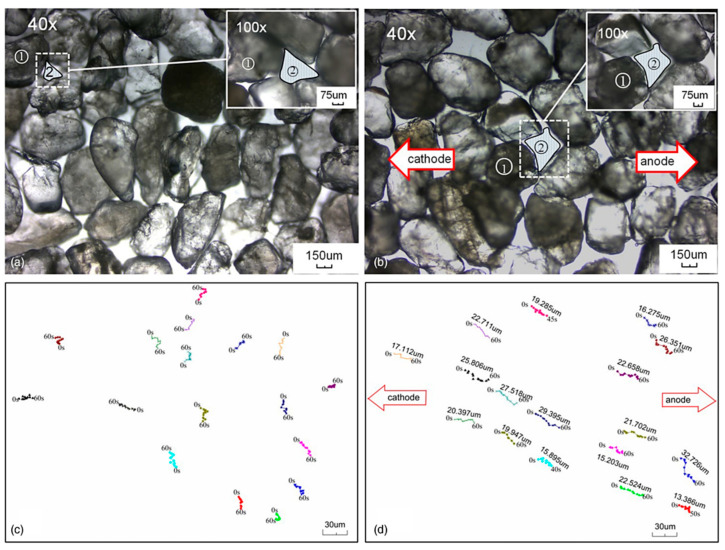
Bacterial movement in the porous media (①—sand matrix; ②—porous media): (**a**) Treated without electrokinetic, magnified at 40× and 100× under an optical microscope; (**b**) Treated with electrokinetic, magnified at 40× and 100× under an optical microscope; (**c**) Treated without electrokinetic, magnified at 200× under an fluorescent microscope; (**d**) Treated with electrokinetic, magnified at 200× under an fluorescent microscope [66].

Deng et al. [65] introduced different electric potential gradients during the culture of *Sporosarcina pasteurii* to study the effect on its physicochemical properties. The amount of calcium carbonate precipitated under a 0.5 V/cm potential gradient exceeded the other groups (Figure 4a). Compared with the untreated bacterial solution, the pH increases under the effect of the weak electric field (Figure 4b) and the alkaline environment is more suitable for bacterial growth, in which the OD_600_ (Figure 4c) and the bacteria liquid enzyme activity (Figure 4d) are significantly increased. Shao et al. [66] conducted a horizontal one-dimensional bio-grouting test in a sand column under a 0.1 V/cm DC field and found that the application of a DC field could not only increase the movement speed of bacteria in the porous medium, but also regulate the migration of bacteria in one direction (Figure 5), which led to a redistribution of the suspended bacteria within the column. Meanwhile, the DC electric field could convert some of the suspended bacteria moving in the soil into attached bacteria, which led to a homogeneous distribution of bacteria, thus improving the homogeneity of calcium carbonate precipitation. Deng et al. [67] utilized bio-grouting combined with electroosmosis to reinforce uranium tailings. Electroosmosis not only improves the migration of polar ions in uranium tailings, but the weak electric field can also change the permeability of bacterial cell membranes, which stimulates the proliferation of bacteria and provides more nucleation sites. The highest content of calcium carbonate was obtained at 0.5 V/cm, and the crystal structure of calcium carbonate showed a strong bonding, which led to a special cementing effect.

#### 3.1.2. Ultraviolet Radiation Mutagenesis

Ultraviolet light has a strong damaging effect on microorganisms, but microorganisms have a strong self-repairing function. This ability to adapt to the environment ensures that microorganisms maintain their species characteristics even if they live in intense radiation. The differences in microbial cell membrane structure, membrane composition, cell inclusions, microbial size, and growth conditions will cause differences in the sensitivity of microorganisms to ultraviolet radiation. This difference provides an opportunity for the use of ultraviolet radiation. The mechanism by which ultraviolet light enhances the performance of the target strain is to use the difference in the sensitivity of microorganisms to ultraviolet light to change the ecosystem in which they are located and cause changes in characteristics, resulting in improved target capabilities. UV mutagenesis has been proven to be an effective bacterial modification technique, which has many advantages such as not involving many safety issues, easy operation, a high mutation rate, and large mutation amplitude [68].

Achal et al. [68] obtained a phenotypic mutant of *Sporosarcina pasteurii*, named Bp M-3 by UV irradiation. Bp M-3 had the highest urease activity and calcite production, as well as a higher production of extracellular polymeric substances and biofilms than the other isolates, and may provide a useful strategy as a sealing agent for filling the gaps or cracks and fissures in any construction structures. Xu et al. [69] used UV mutagenesis on bacteria (named CX21) isolated from soil in Fujian Province, and the urease activity of the bacterium (YB7) was up to 0.72 ms/min, which was 2.18 and 1.33 times higher than that of *Sporosarcina pasteurii* and CX21, respectively. The results indicate that it is feasible to utilize UV-induced bacteria for tailings consolidation to mitigate the associated storage safety risks. Zhang et al. [70] used UV mutagenesis to improve the urease-producing strain, and the morphology of the strain was changed before and after mutagenesis. After mutagenesis, the strain was a short elliptical column with a reduced size, which was the reason for the precipitated small and dense calcium carbonate crystals, thus improving the strength of the bio-reinforced sand column.

#### 3.1.3. Ultrasound

Under the stimulation of ultrasound, the composition and structure of bacterial microorganisms will change. Low-intensity ultrasound can effectively improve bacterial activity. The principle is that ultrasound affects the physical morphology of the cell membrane, which greatly reduces the resistance of the enzyme to pass through the cell membrane, and the intracellular biological enzyme is therefore easier to discharge out of the cell, so that the enzyme is has more contact with the reaction reagent, and, therefore, the reaction efficiency is higher. At the same time, under the influence of the ultrasonic cavitation effect, the shearing effect of cavitation bubbles can accelerate the contact time between the substrate and the enzyme, so that the reaction product is far away from the enzyme activity center in time, creating opportunities for new substrates to contact the enzyme and thereby improving the enzyme catalytic efficiency. However, when the ultrasonic intensity is too high, the biological enzyme will be denatured and inactivated, making it lose its catalytic function [71]. Liu et al. [72] utilized the principle of the effect of ultrasound on bacteria and used *Sporosarcina pasteurii* treated with ultrasound for the MICP process, and they found that the calcium carbonate precipitated in the aqueous solution and in the sand column was increased by 28.5% and 35.6%, respectively, and the unconfined compressive strength of the sand samples after reinforcement was 1.25 MPa, which is 91.6% higher than that of the control group.

As mentioned above, ultrasound can also be used for cell fragmentation to obtain urease for the EICP process in isolation from the bacteria. Whether it is an electric field, ultraviolet mutagenesis, or ultrasound, the improvement in the bacterial performance depends on the applied intensity of the scheme used. Excessive intensity will always cause bacterial death, and appropriate intensity is conducive to the MICP process. The physical scheme of bacterial modification described in this paper has a certain guiding role in improving the application of MICP in practical engineering. In the future, new physical methods still follow this basic principle. Finding the appropriate treatment intensity is the key factor to successfully improving bacterial performance.

### 3.2. Chemical Method

The use of chemistry to enhance bacterial properties is mainly achieved through the addition of additives, and the use of chemical additives has two main effects on MICP, including changing the properties of the bacteria and the morphology of the formed CaCO_3_ [73].

**Figure 6:**
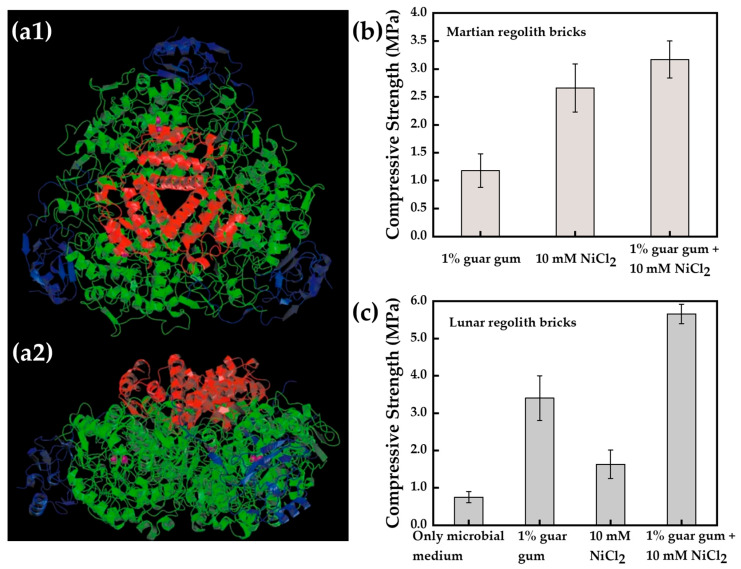
(**a**) Ribbon diagram of the (αβγ)3 heterotrimer of urease: (**a1**) view down the crystallographic threefold axis; (**a2**) view from the side. The green, blue, and red ribbons represent, respectively, the α, β, and γ subunits. The magenta spheres in the α subunits are the nickel ions of the active center [74]; (**b**) Compressive strengths of martian bricks for different treatments (Specimens treated with microbiological media only cannot be consolidated and therefore do not indicate strength); (**c**) Compressive strengths of lunar bricks for different treatments ((**b**,**c**) adapted from Dikshit et al. [77]).

Studies have shown that urease is a nickel-containing oligomeric enzyme composed of three distinct subunits, with two nickel atoms distributed in different active sites of the urease macromolecular structure (Figure 6(a1,a2)), thus affecting urease activity and the structural integrity of urease [74]. Through circular dichroism and nuclear magnetic resonance spectroscopy, Won et al. [30,75] found that the two conserved histidine-containing side chains at the C-terminus of the ureE accessory protein of *Sporosarcina pasteurii* were the main ligands of nickel ions. Through the study of ureE, it can be found that nickel ions play an important role in urease activity. Carlsson et al. [76] used density functional theory to calculate the active site model of urease, indicating that urea was initially bound to the nickel ion of the active site through its oxygen atom, and then hydrolyzed urea. In practical applications, Dikshit et al. [77] used urease-producing bacteria to induce the MICP process in two simulated soils and used NiCl_2_ as a biocatalyst to increase urease activity, thereby improving the compressive strength of bio-bricks (Figure 6b,c). Slurries mixed only with *S. pasteurii* media were not robust enough for uniaxial measurements in the MICP-induced consolidation of martian regolith bricks, whereas the mean compressive strength of the reinforced brick samples obtained by adding 10 mM NiCl_2_ was about 2.67 MPa. In the MICP-induced consolidation experiments of lunar regolith bricks, the compressive strength of the specimens with NiCl_2_ was increased by 117% compared to the specimens that were consolidated only with the microbial medium, and the compressive strength of the specimens could be increased by a maximum of 653% when NiCl_2_ and guar gum were added to the specimens at the same time.

In addition to nickel ions, other metal ions can have an effect on bacteria and the MICP process. Sun et al. [78] found that high concentrations of Mg^2+^ inhibited bacterial urease activity, but Mg^2+^ promoted urease activity when Mg^2+^ did not exceed 1.5 M. Lv et al. [79] found that the addition of Mg^2+^ to the MICP cementing solution can slow down the rate of the reaction consumption of calcium ions, and the bio-cemented samples can precipitate 23.9% carbonate precipitate with a maximum unconfined compressive strength of 6.2 MPa when the Mg^2+^ is 0.5 M. At the same time, the addition of Mg^2+^ can change the morphology of calcium carbonate and promote aragonite generation [80]. In summary, the effect of magnesium ions on the MICP process is complex, with magnesium ions affecting not only the bacterial urease activity but also the morphology of the formed CaCO_3_. At the same time, the decomposition of urea by bacterial urease promotes the generation of magnesium carbonate precipitate from Mg^2+^. This interaction process was also found in the addition of Al^3+^ to the MICP cementing solution. Wei et al. [9,60] added a small amount of Al^3+^ to the MICP cementing solution and found that it could lower the pH of the solution, which in turn affects the bacterial urease activity, whereas the MICP reaction raises the pH of the solution and precipitates the Al^3+^ as Al(OH)_3_, which is capable of changing the distribution pattern of calcium carbonate in the soil, thus dramatically increasing the UCS of the reinforced sand. The interaction of metal ion–bacterial urease–precipitate is an extremely complex process, especially the effect of metal ions on mineralized bacteria, which still needs further study.

In addition to inorganic metal ions, other chemical additives have been used to treat bacteria. Li et al. [81] treated *Sporosarcina pasteurii* by nitrosoguanidine mutagenesis to obtain a high urease-producing mutant strain, which showed a 207% increase in activity over the pre-treatment strain. Xiao et al. [82] used Triton X-100 to increase the cell membrane permeability of *Sporosarcina pasteurii*, which promoted the hydrolysis of urea and increased the rate of calcium carbonate generation, while the structure of calcium carbonate generated by the bacteria as the nucleation site was more dense, and effectively improved the MICP reinforcement efficiency. Xu et al. [83,84] introduced the urease inhibitor NBPT into the MICP reaction, which could prolong the time of urea hydrolysis and calcium carbonate precipitation, solving the problem of localized clogging and thus improving the reinforcement effect. Gu et al. [85] added 0.05% glutaraldehyde and 1% silk protein before the MICP treatment, and the bacterial activity was stimulated to a certain extent under the weak toxic environment of glutaraldehyde, while silk protein provided more nucleation sites for the precipitation of calcite, which not only significantly reduced the porosity of the specimen, but also effectively precipitated the calcite between the sand grains, thus significantly enhancing the MICP reinforcement effect.

### 3.3. Biological Method

#### 3.3.1. Hybrid Bacteria

It has been demonstrated that there is a synergistic effect between microorganisms [86], which is a breakthrough in the engineering application of microbial-induced mineralization technology. Compared to single bacteria, co-cultured bacteria showed better performance [87,88].

Gat et al. [89] performed MICP experiments in specific co-cultures of ureolytic and non-ureolytic bacteria, and CaCO_3_ precipitated more rapidly than the control group, despite the lower pH and carbonate ion concentration of the mixed cultures. *Bacillus subtilis* exhibited a much higher growth rate than *Sporosarcina pasteurii*, resulting in a higher density of bacterial cells in the mixed cultures, which facilitated CaCO_3_ precipitation by providing additional nucleation sites. Harnpicharnchai et al. [90] mixed four bacterial species for the MICP reaction, and the remaining three strains, except *Sporosarcina pasteurii*, precipitated a large amount of CaCO_3_ in the absence of urea, revealing the potential of utilizing these strains for MICP without the need of urea. At the same time, the mixed cultures precipitated about 1.4 times higher CaCO_3_ than that obtained from *Sporosarcina pasteurii* alone. Jang et al. found that bacteria co-cultured with alkaliphilic and alkali-resistant strains showed better performance than single-strain bacteria, with higher resistance to pH and harsh environments, and could promote biofilm formation and CaCO_3_ precipitation. Compared with pure bacteria, the microbial community (mixed bacteria) composed of multiple mineralized microorganisms has better adaptability to environmental conditions, their mineralization efficiency is higher, and the cultivation cost is only about one-third of that of pure bacteria [91].

#### 3.3.2. Genetically Engineered Bacterium

Genetically engineered bacterium is a bacterium in which a target gene is introduced into a bacterium to make it express and produce the desired protein. The core technology of genetic engineering is the recombination technology of DNA, which is to extract a certain amount of DNA or synthetic DNA from an organism, recombine it with plasmid or phage as a vector in vitro, and then transfer it to another organism so as to change the genetic traits of the organisms [92]. Genetically engineered bacteria should have the following conditions: (1) the fermentation product has high concentration, conversion, and yield; (2) the strain can utilize commonly used carbon sources and can have continuous fermentation; (3) the strain is not pathogenic or endotoxin-producing; (4) the metabolic control is easy to carry out; and (5) it is capable of the recombination of DNA and is stable.

**Figure 7:**
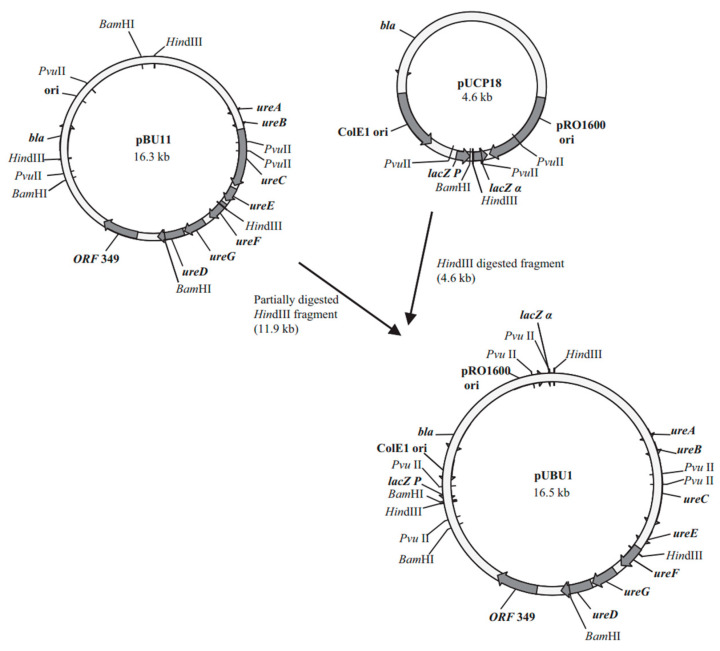
Construction of plasmid pUBU1 [94].

At present, a large number of studies on the composition and expression regulation of urease genes have been conducted in the literature, which paves the way for DNA recombination in urease-based bacteria. Connolly et al. [93] introduced the urease gene of *S. pasteurii* into *P. aeruginosa* and *E. coli*, which can express green fluorescent protein GFP. The urease genes ureDABC and ureFG were ligated into the vector pJN105 to construct two new model organisms. Through the imaging of GFP under a fluorescence microscope, it is convenient to study the mechanism of the temporal and spatial distribution of microorganisms and calcium carbonate during mineralization. Bergdale et al. [94] cloned the urease gene sequence in the pBU11 plasmid into the vector pUCP18, as shown in Figure 7. The newly constructed plasmid pUBU1 was transformed into two strains of Pseudomonas aeruginosa to develop a recombinant that can induce calcite precipitation in addition to producing an EPS. The rate of CaCO_3_ precipitation induced by recombinant Pseudomonas was comparable to that of *Sporosarcina pasteurii*. Scanning electron microscopy showed that there was a complex of CaCO_3_ crystals and EPS layers around the cells. Li et al. [95] prepared a composite biofilm with the function of microbial-induced calcium carbonate precipitation by introducing recombinant urease, gene programming curli, and covalent conjugated xanthan gum into engineering E.coli. The composite biofilm can be used to reinforce loose sand. In the process of bio-cementation, urease provides biomineralization ability, recombinant curli enhances bacterial adhesion, xanthan gum enhances bacterial activity and cementation strength, and the compressive strength of bio-cemented sand can reach 3.14 MPa.

Whether it is a hybrid or genetically engineered bacterium, the use of biological methods to improve MICP is different from physical and chemical methods. Physical and chemical methods only target a single bacterium, while biological methods introduce new bacteria or genes based on the research of *Sporosarcina pasteurii*, and their combined functions with *Sporosarcina pasteurii* can respond to different engineering needs. However, in the field of MICP, the research on hybrid and genetically engineered bacteria is still in its infancy, and there is a great potential for the application of different bacteria or gene combinations.

## 4. New Approaches to Address the Challenges in MICP

### 4.1. Improvements to Address Adverse Environmental Factors

**Figure 8:**
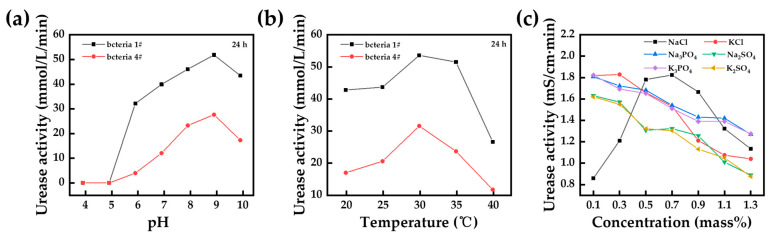
Effect of different environmental factors on bacterial activity: (**a**) pH; (**b**) temperature; (**c**) salt concentration ((**a**,**b**) adapted from Dong et al. [100]; (**c**) adapted from Han et al. [101]).

When using bacterial suspensions or urease solutions for biomineralization, the effect of environmental factors on them should be considered. Most of the literature has shown that a pH in the range of 6 to 9 does not significantly affect the rate of urease hydrolysis by *Sporosarcina pasteurii* suspensions [96,97,98]. Meanwhile, temperature is one of the key factors affecting the growth and reproduction of microorganisms. The optimum temperature of the MICP process is similar to the optimum temperature of urease-producing microorganisms, which is about 20~40 °C [25,99]. Dong et al. [100] compared the urease activity of *Sporosarcina pasteurii* (named bacteria 1# and bacteria 4#, respectively) extracted from two different areas after 24 h at a different pH and temperature, and the results are shown in Figure 8a,b. Although there were differences in the urease activity between the two bacteria, the pattern of change was consistent under different pH and temperature conditions. The bacteria had the highest activity at pH 9 and reached the maximum value at a temperature of 30 °C.

In addition to pH and temperature, certain factors in specific environments can affect bacterial reproduction and activity. Han et al. [101] investigated the effect of different salt solutions (NaCl, KCl, Na_3_PO_4_, and Na_2_SO_4_) on the survival characteristics of *Bacillus Pasteurii*, as shown in Figure 8c. The optimal NaCl concentration for bacterial survival was 0.3~0.9 mass%, and excess K+ (0.3 mass%) may be harmful or even lethal to the cells. In addition, excess PO_4_^3−^ and SO_4_^2−^ inhibited the multiplication of *Bacillus Pasteurii* and reduced urease activity.

Environmental factors will affect the growth and reproduction of bacteria, but in a specific environment, some microorganisms can adapt to the environment or mutate to become the dominant flora, which is a natural domestication process. The same effect can be obtained through artificial domestication by gradually adding materials or substrates that simulate the corresponding environment into the bacterial culture medium, allowing the bacteria to gradually adapt to the culture environment and thus domesticate the microbial groups and species that are tolerant to such materials or substrates. Shi et al. [102] domesticated *Bacillus megaterium* in order to improve its growth performance at different pH values, reproductive performance, and urease activity under an alkaline environment. It was found that the increase in urea could significantly improve the propagation properties, urease activity, and calcium production rate of microorganisms in alkaline environments, and the method could effectively solve the problem of insufficient calcium carbonate precipitation under strong alkaline conditions. The alkaline domesticated *Bacillus megaterium* could reduce the permeability properties of concrete better than the undomesticated *Bacillus megaterium*, and the strength of its repaired specimens was increased by 2.1 times. Sun et al. [103] studied the effect of urea on the process of the microbial cementation of sandy soil and found that the addition of 5–20 g of urea to the culture solution had a promotional effect on urease activity and an inhibitory effect on the growth of the strain, which was caused by the alkaline environment produced by the decomposition of urea, but the addition of urea after sterilization was less inhibiting and the method had the advantage of rapid cementation. Using this property, Sun et al. [104] added urea to the nutrient solution to domesticate *Bacillus megaterium* under low-temperature conditions, and the low precipitation yield at low temperatures was caused by the double inhibition of the growth and reproduction of *Bacillus megaterium* and the urease activity under low-temperature conditions; the MICP reaction of the domesticated bacteria could significantly improve the calcium carbonate precipitation yield, which could effectively solve the problem of insufficient calcium carbonate precipitation under low-temperature conditions. The domestication of the bacteria has led to wider applications in practical engineering, such as in the reinforcement of MICP sandy soils in marine engineering. Peng et al. [105] found that the seawater environment adversely affected the UCS of MICP-reinforced calcareous sand compared to freshwater environment. In order to solve the inhibitory effect of seawater environment on *S. pasteurii*, Xiao et al. [106] designed a multi-gradient artificial domestication culture experiment of *S. pasteurii* in an artificial seawater environment. After five gradients of domestication, *S. pasteurii* had good adaptability, the bacterial cells became smaller, and the carbonate (calcium and magnesium carbonate) crystals generated in the seawater environment were smaller and denser, which could better fill the pores of calcareous sand particles and cement adjacent calcareous sand particles. In terms of solid waste resource utilization, Guo et al. [107] obtained *Sporosarcina pasteurii* with special resistance through strain domestication, which can tolerate both solid waste gangue leachate and 1 M urea, and found that biomineralization greatly improved the microstructure of solid waste gangue particles.

**Figure 9:**
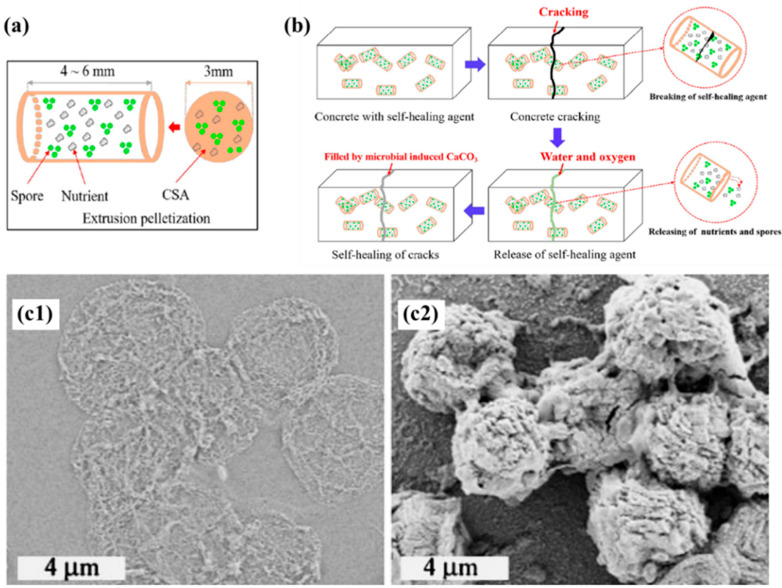
(**a**) Schematic diagram of microbial pellets [109]; (**b**) self-healing schematic diagram of microbial pellets [109]; (**c**) SEM images of polyelectrolyte capsule (**c1**) before CaCO_3_ formation and (**c2**) after CaCO_3_ formation [110].

Natural domestication and artificial domestication are carried out by altering bacterial adaptations to enable them to survive and reproduce in specific environments, while some scholars have improved the environment in order to achieve conditions suitable for bacterial survival. Farmani et al. [108] reduced the pH value of concrete by replacing 20% cement with silica fume. After microbial mineralization repair, compared with normal samples, the compressive strength of silica fume-modified mortar specimens was always significantly higher than that of ordinary mortar specimens. For the harsh environment inside the concrete material, the use of carrier materials to immobilize microorganisms has been proposed in a large number of studies, and this method not only avoids direct contact between microorganisms and the alkaline environment, but also provides a stable living environment for the growth and metabolism of microorganisms [109,110,111]. Su et al. [109] extruded microorganisms, nutrients, and carriers into a microsphere (Figure 9a) to develop a novel microbial self-repairing system (Figure 9b), which is well protected against microbial spores and has good applications in the field of concrete self-repairing. Alexei et al. [110] proposed a new biomimetic method for CaCO_3_ synthesis inside micron-sized polyelectrolyte capsules, in which the reaction of the bacterial precipitation of CaCO_3_ occurs inside the capsule and the precipitate formed can completely fill the inside of the capsule (Figure 9(c1,c2)). Seifan et al. [111] synthesized magnetic iron oxide nanoparticle ions in an aqueous solution, the ions were adsorbed on the surface of the bacterial cells due to electrostatic forces, and the adsorbed magnetic ions could penetrate through the cell wall of the bacteria, thus protecting the bacteria from the harmful environment. There are many kinds of carriers for immobilizing microorganisms, such as diatomite [112], expanded perlite [113], microcapsules [114], and hydrogels [115]. In addition to protecting bacteria, different carriers also have unique properties, and their adaptability to different environments is also different. Appropriate immobilized carriers should be selected for practical engineering.

### 4.2. One-Phase Injection Method to Improve Homogeneity of Bio-Reinforced Sand

In the traditional MICP scheme, bio-flocculation occurs immediately when the bacterial solution is mixed with the cementing solution. In most of the literature, the bacterial solution (or enzyme solution) is injected first, followed by the cementing solution, and the distribution of calcium carbonate crystals in the bio-cemented sandy soil obtained by this method is not uniform because the subsequently injected cementing solution can easily wash away the bacterial cells or enzymes in the near area of the injection point, especially those with poor adsorption capacity [41,116]. The uniformity of reinforcement becomes an urgent problem for MICP technology.

**Figure 10:**
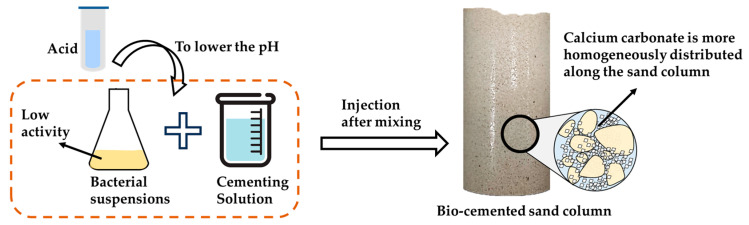
A Low-pH one-phase injection method in MICP.

A lower pH affects the urease activity of the bacteria, but this does not mean the death of the bacteria. At a lower pH, the bacteria can still secrete urease, and as the urease hydrolyzes urea, the pH value begins to rise. This process can also be used to improve the properties of MICP-reinforced soil. Cheng et al. [61] proposed a low-pH one-phase injection method, as shown in Figure 10. The principle is to mix the bacteria and cementing solution and to use hydrochloric acid to reduce the activity of bacterial urease, so that it cannot quickly produce biological flocculation precipitation. As the bacteria decompose urease, the pH increases and the calcium carbonate precipitation begins to deposit. In this process, the precipitation of calcium carbonate will have a lag period. Using this lag period, the mixture of bacteria and the cementing solution can be injected into the soil together. The soil treated by this scheme not only improves the uniformity, thus improving the overall strength, but also has less ammonia emission, so as to obtain more efficient and environmentally friendly effects.

On the basis of Cheng’s study [61], numerous scholars have further investigated the low-pH one-phase injection method. Yang et al. [117] used acetic acid instead of hydrochloric acid to reduce the pH of the all-in-one solution, which was able to prolong the time of calcium carbonate lag precipitation, and the reinforced sand column was more uniform. Yu et al. [118] combined one-phase MICP injection with MISP (microbial-induced struvite precipitation) technology, which was able to further reduce ammonia release from MICP and obtain cleaner bio-cement. Lai et al. [119] investigated the reinforcement of different sands by the MICP low-pH one-phase injection method, and found that the environmental pH varies in different sands, which affects the reinforcement effect, and the optimal reaction pH of different bacterial solution concentrations should be adjusted according to the soil properties in order to achieve the best results. Cui et al. [41,120] verified the feasibility of this method for EICP application, and the low-pH one-phase method significantly improved the calcium conversion efficiency and the uniformity of calcium carbonate distribution in sand samples compared with the traditional two-phase EICP method.

**Figure 11:**
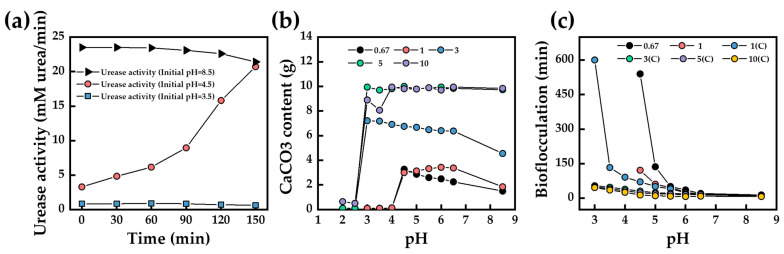
(**a**) Influence of pH changes on urease activity (0.67 × 108 cells/mL bacterial solution, adapted from Lai et al. [119]); (**b**) CaCO_3_ content after 7 days of reaction (0.67, 1, 3, 5, and 10 indicate 0.67 × 10^8^, 1 × 10^8^, 3 × 10^8^, 5 × 10^8^, and 10 × 10^8^ cells/mL bacterial solutions, respectively); (**c**) bio-flocculation generation time (0.67, 1, 3, 5, and 10 indicate 0.67 × 10^8^, 1 × 10^8^, 3 × 10^8^, 5 × 10^8^, and 10 × 10^8^ cells/mL bacterial solutions, respectively; C: centrifugation, (**b**,**c**) adapted from Lai et al. [121]).

In the low-pH one-phase injection method, the pH and nature of the bacterial urease are key factors. Bacterial urease is inactivated when the initial pH is too low (Figure 11a) [119], and a significant decrease in efficiency is observed when the initial pH of the all-in-one solution is less than 4 [61]. When the bacterial concentration is too low, it reduces the cementing solution utilization (Figure 11b), while when the bacterial concentration is too high, it shortens the lag period of calcium carbonate precipitation (Figure 11c) [121]. Lai et al. [122] explored the effect of the initial pH on the low-pH one-phase injection method. The initial pH was in the range of 4–7, and the pH had little effect on the calcium carbonate yield and its distribution along the height of the sand column, but the initial pH affects the morphology of the calcium carbonate crystals and the degree of crystallinity, thus affecting the strength of the treated sand, and the treated sand obtained the maximum unconfined compressive strength at an initial pH of 5.5. Zhang et al. [123] proposed a method to enhance the curing efficiency of the low-pH one-phase injection method with a low bacterial concentration and found that, compared with a one-time injection of a high bacterial concentration, a batch injection of a low bacterial concentration not only solves the problem of the low utilization of the cementing solution, but also produces more and larger calcium carbonate crystals, which can significantly improve the unconfined compressive strength of cured sand columns.

The low-pH environment, although adversely affecting the bacterial urease, can also provide a new MICP treatment method, which is the low-pH one-phase injection method. This method not only improves the homogeneity of calcium carbonate distribution in soils and increases the strength of bio-reinforced soils, but also reduces the emission of ammonia as a byproduct of MICP, which provides a new way of thinking to improve the efficiency of reinforcement, green construction, and thus promote the development of MICP technology.

**Figure 12:**
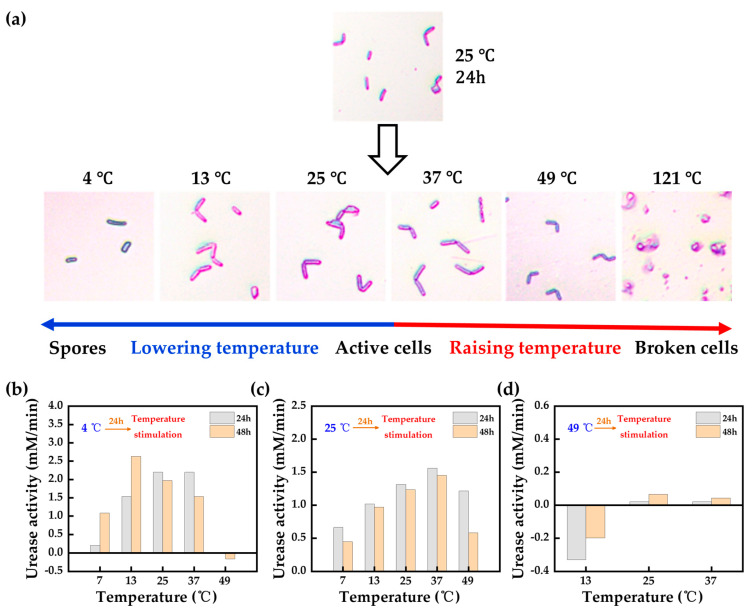
(**a**) The cell shape changes in bacteria under different temperature stimulation; (**b**) the growth and mineralization of bacteria when the temperature rises from 4 °C to different temperatures; (**c**) the growth and mineralization of bacteria when the temperature changes from 25 °C to different temperatures; (**d**) the growth and mineralization of bacteria when the temperature changes from 49 °C to different temperatures (adapted from Li et al. [124]).

The low-pH one-phase injection method can improve the homogeneity of the bio-reinforced soil, thus increasing the strength, which can be realized by using a low pH to temporarily reduce the urease activity and delay the calcium carbonate precipitation time, while the low temperature can also reduce the urease activity to achieve the same effect. Li et al. [124] found that *Sporosarcina pasteurii* are in a spore state at low temperatures, while the cells are broken down at high temperatures (Figure 12a). Through laboratory experiments, it was found that it was possible to achieve an increase in activity when the temperature increased from low temperatures (Figure 12b), whereas, when the temperature was lowered from higher temperatures, the effect was not satisfactory (Figure 12d). The optimal temperature range for the growth of *Sporosarcina pasteurii* is 7~37 °C. In this range, increasing the culture temperature from low temperatures can enhance the microbial activity and urease activity (Figure 12b,c).

To avoid clogging, Kalantary et al. [125] inhibited bacterial activity and delayed CaCO_3_ precipitation by lowering the reaction temperature of the mixture to 3 °C prior to injection. The experimental results showed that the maximum compressive strength obtained from a one-phase injection at room temperature was about 80 kPa, while the compressive strength of the treated samples was increased to 230 kPa by lowering the temperature of the bacterial suspension and the reactant solution before injection. Liu and Xiao et al. [126,127,128] proposed a temperature-controlled one-phase MICP reinforcement method, which enables the MICP reaction to be maintained in a low-temperature environment for a period of time, at which time the urease activity is low, and the bacterial suspension can be injected into the soil by mixing with the cementing solution, where the precipitation begins to be generated when the ambient temperature rises. Compared with the room temperature two-phase method, the temperature-controlled one-phase method can obtain a more homogeneous distribution of calcium carbonate in the soil, which can enhance the reinforcement effect.

## 5. Perspectives

Overall, the research on MICP technology has made promising progress. The excellent properties of bacteria, urease, and precipitated calcium carbonate have made it attractive in different engineering fields. Although biomineralization does not produce CO_2_ like cement does, the decomposition of urea is accompanied by the production of ammonia. The long-term effects of bacteria and their products on the environment are still worthy of attention. On the other hand, compared to cement, MICP technology is more costly, including the large-scale cultivation of bacteria and the consumption of cementing materials, which limits the application of MICP in practice. How to apply MICP technology in practice efficiently, economically, and environmentally is still an urgent issue to be solved in this field in the future.

In this review, we propose some improvement schemes that can improve MICP efficiency and reduce ammonia emissions, but these methods still have room for further improvement. From a genetic point of view, research on the use of genetic engineering techniques to improve *Sporosarcina pasteurii* is at a preliminary stage. The cultivation of ‘super engineering bacteria’, based on *Sporosarcina pasteurii*, or urease-expressing bacteria by embedding gene fragments is an attractive field that can dramatically improve performance in special environments. From a molecular point of view, synergy between different bacteria can be utilized to meet practical needs. The in-depth study of the performance of different bacteria and the reasonable regulation of the synergy between different bacteria can better meet the rational use of resources and achieve the purpose of economic and environmental protection. In addition to the synergy between different bacteria, the study of the interaction between bacteria and the generated minerals can further explain the mechanism of MICP reinforcement and adsorption. The recent development of microfluidics combined with MICP technology provides new ideas to observe the MICP process from a microscopic molecular point of view, as well as new methods for the observation of the behavior of bacteria in different materials [129,130,131]. From a macroscopic point of view, synergies between bacteria and other materials are also aspects of interest. The role of materials can be multifunctional, such as carrier roles, protective roles, etc., as mentioned in this paper. In addition to this, there are also studies on the incorporation of zeolite into the MICP process, where zeolite can absorb the MICP by-product ammonia [132,133]. Exploring new materials to cooperate with MICP can meet different engineering needs. From the perspective of engineering applications, in the application of soil reinforcement in geotechnical engineering, there is still a gap between MICP technology and cement in terms of economy. Reducing the cost of bacterial culture and finding cheap replacements for cementing materials are still the focus of research. However, it is exciting to note that MICP technology has been well adopted in some applications, such as wind erosion control. A large number of field trials have been carried out to support the potential of the technology for practical applications in desertification control [12,134,135]. In general, MICP has great application value in some fields, such as water treatment, heavy metal adsorption, and so on.

In addition to the improvement of bacterial performance, there is still room for MICP to be improved in other aspects, such as the improvement of the cementing solution, the improvement of different treatment methods (e.g., infusion, spraying, stirring, etc.) to cope with the real structures, etc. This paper provides some reference for the subsequent research and application of MICP technology.

## 6. Conclusions

(1) In this paper, we describe the urease composition at the genetic level and review the sources of urease. Urease decomposes urea and induces calcium carbonate precipitation. In MICP, bacteria act as calcium carbonate nucleation sites in addition to secreting urease for the biomineralization process, and their secreted EPS also influence calcium carbonate production. Besides the bio-augmentation method, the MICP process can also be conducted by bio-stimulation as well as extraction and enrichment methods. Different schemes are compared and analyzed from the perspectives of environmental suitability, time cost, environmental dependence, and raw material consumption.
(2) Physical (electric field, UV mutagenesis, and ultrasound), chemical (inorganic and organic additives), and biological (hybrid bacteria and DNA recombination) methods to enhance bacterial performance were summarized, and the performance of bacteria was enhanced in different ways by different treatments.
(3) The effects of different environmental factors on bacterial growth (e.g., pH, temperature, salinity, etc.) are described, and improvement methods are proposed for adverse environmental factors, such as the domestication of bacteria to cope with adverse environments and the protection of bacteria through carriers. Although unfavorable environmental factors can adversely affect bacterial urease, they can also provide new MICP treatment methods; that is, one-phase injection methods based on low temperatures or low pHs, which can significantly improve the efficiency of MICP reinforcement.

## Figures and Tables

**Table 1:** Evaluation of different cultivation methods of bacteria. (Note: The number of ★ represents degree. For example, the methods of Bio-stimulation and Isolation and Enrichment are better at Environmental Suitability than the methods of Bio-augmentation, so they have more ★. The rest of the properties are similar).

Processing Method	Environmental Suitability	Time Cost	Environmental Dependence	Raw Material Consumption
Bio-augmentation	★	★★	★	★★
Bio-stimulation	★★★	★	★★★	★
Isolation and Enrichment	★★★	★★★	★★★	★★★

## Data Availability

No new data were created or analyzed in this study. Data sharing is not applicable to this article.

## References

[1] Griesshaber E., Schmahl W. (2021). Special Issue on Biomineralization: From Cells to Biomaterials. Acta Biomater..

[2] Liu H.L. (2024). Biogenic construction: The new era of civil engineering. Biogeotechnics.

[3] Wang Y., Sun X., Miao L., Wang H., Wu L., Shi W., Kawasaki S. (2024). State-of-the-art review of soil erosion control by MICP and EICP techniques: Problems, applications, and prospects. Sci. Total Environ..

[4] Chu J., Stabnikov V., Ivanov V. (2012). Microbially Induced Calcium Carbonate Precipitation on Surface or in the Bulk of Soil. Geomicrobiol. J..

[5] Dong Z.-H., Pan X.-H., Zhu C., Tang C.-S., Lv C., Liu B., Wang D.-L., Li H., Cheng Y.-J., Shi B. (2024). Bio-mediated geotechnology and its application in geoengineering: Mechanism, approach, and performance. Environ. Earth Sci..

[6] Ishara S., Anand R., Parihar A., Reddy M.S., Goyal S. (2024). Suitability and Challenges of Biomineralization Techniques for Ground Improvement. Int. J. Environ. Res..

[7] Jiang L., Xia H., Wang W.J., Zhang Y., Li Z. (2023). Applications of microbially induced calcium carbonate precipitation in civil engineering practice: A state-of-the-art review. Constr. Build. Mater..

[8] Badiee H., Sabermahani M., Javadi A.S., Bouazza A. (2021). Comparison of Microbially Induced Carbonate Precipitation with Ordinary Portland Cement Producing Macroporous Pervious Concrete. J. Mater. Civ. Eng..

[9] Wei R.J., Peng J., He J., Li L.L., Jiang Z., Tang J.H. (2024). Effects of adding aluminum ion flocculant on MICP reinforcement of sand. Acta Geotech..

[10] Jiang N.-J., Wang Y.-J., Chu J., Kawasaki S., Tang C.-S., Cheng L., Du Y.-J., Shashank B.S., Singh D.N., Han X.-L. (2022). Bio-mediated soil improvement: An introspection into processes, materials, characterization and applications. Soil Use Manag..

[11] Cui M.-J., Zheng J.-J., Zhang R.-J., Lai H.-J., Zhang J. (2017). Influence of cementation level on the strength behaviour of bio-cemented sand. Acta Geotech..

[12] Li S.H., Li C., Yao D., Wang S. (2020). Feasibility of microbially induced carbonate precipitation and straw checkerboard barriers on desertification control and ecological restoration. Ecol. Eng..

[13] Dagliya M., Satyam N., Sharma M., Garg A. (2022). Experimental study on mitigating wind erosion of calcareous desert sand using spray method for microbially induced calcium carbonate precipitation. J. Rock Mech. Geotech. Eng..

[14] Jiang L., Li P., Wang W., Zhang Y., Li Z. (2024). A self-healing method for concrete cracks based on microbial-induced carbonate precipitation: Bacteria, immobilization, characterization, and application. J. Sustain. Cem.-Based Mater..

[15] Qian C., Zheng T., Zhang X., Su Y. (2021). Application of microbial self-healing concrete: Case study. Constr. Build. Mater..

[16] Garg R., Garg R., Eddy N.O. (2023). Microbial induced calcite precipitation for self-healing of concrete: A review. J. Sustain. Cem.-Based Mater..

[17] Guo L., Wang B., Guo J., Guo H., Jiang Y., Zhang M., Dai Q. (2024). Experimental study on improving hydraulic characteristics of sand via microbially induced calcium carbonate precipitation. Geomech. Energy Environ..

[18] Xiao P., Liu H., Xiao Y., Stuedlein A.W., Evans T.M. (2018). Liquefaction resistance of bio-cemented calcareous sand. Soil Dyn. Earthq. Eng..

[19] Han Z., Zhang J., Bian H., Yue J., Xiao J., Wei Y. (2024). Study on Liquefaction-Resistance Performance of MICP-Cemented Sands: Applying Centrifuge Shake Table Tests. J. Geotech. Geoenviron. Eng..

[20] Mu B., Gui Z., Lu F., Petropoulos E., Yu Y. (2021). Microbial-Induced Carbonate Precipitation Improves Physical and Structural Properties of Nanjing Ancient City Walls. Materials.

[21] Wu H.R., Shi J.Q., Xiao Y., He J.L., Chu J. (2023). Microbial mineralization: A promising approach for stone cultural relics restoration. Biogeotechnics.

[22] Cui J., Xie Y., Sun T., Chen L., Zhang W. (2021). Deciphering and engineering photosynthetic cyanobacteria for heavy metal bioremediation. Sci. Total Environ..

[23] Landa-Marban D., Tveit S., Kumar K., Gasda S.E. (2021). Practical approaches to study microbially induced calcite precipitation at the field scale. Int. J. Greenh. Gas Control.

[24] Nething C., Smirnova M., Groening J.A.D., Haase W., Stolz A., Sobek W. (2020). A method for 3D printing bio-cemented spatial structures using sand and urease active calcium carbonate powder. Mater. Des..

[25] Tang C.-S., Yin L.-Y., Jiang N.-J., Zhu C., Zeng H., Li H., Shi B. (2020). Factors affecting the performance of microbial-induced carbonate precipitation (MICP) treated soil: A review. Environ. Earth Sci..

[26] Liu Y., Ali A., Su J.-F., Li K., Hu R.-Z., Wang Z. (2023). Microbial-induced calcium carbonate precipitation: Influencing factors, nucleation pathways, and application in waste water remediation. Sci. Total Environ..

[27] Erdmann N., Strieth D. (2023). Influencing factors on ureolytic microbiologically induced calcium carbonate precipitation for biocementation. World J. Microbiol. Biotechnol..

[28] Pei D., Liu Z.-M., Hu B.-R., Wu W.-J. (2020). Progress on Mineralization Mechanism and Application Research of *Sporosarcina pasteurii*. Prog. Biochem. Biophys..

[29] Liu X., Zhang Q., Zhou N., Tian Y. (2017). Expression of an Acid Urease with Urethanase Activity in *E-coli* and Analysis of Urease Gene. Mol. Biotechnol..

[30] Won H.S., Lee B.J. (2004). Nickel-binding properties of the C-terminal tail peptide of *Bacillus pasteurii* UreE. J. Biochem..

[31] Lee Y.H., Won H.S., Lee M.H., Lee B.J. (2002). Effects of salt and nickel ion on the conformational stability of *Bacillus pasteurii* UreE. FEBS Lett..

[32] Mulrooney S.B., Ward S.K., Hausinger R.P. (2005). Purification and properties of the *Klebsiella aerogenes* UreE metal-binding domain, a functional metallochaperone of urease. J. Bacteriol..

[33] Tobler D.J., Cuthbert M.O., Phoenix V.R. (2014). Transport of *Sporosarcina pasteurii* in sandstone and its significance for subsurface engineering technologies. Appl. Geochem..

[34] Saif A., Cuccurullo A., Gallipoli D., Perlot C., Bruno A.W. (2022). Advances in Enzyme Induced Carbonate Precipitation and Application to Soil Improvement: A Review. Materials.

[35] Das N., Kayastha A.M., Srivastava P.K. (2002). Purification and characterization of urease from dehusked pigeonpea (*Cajanus cajan* L.) seeds. Phytochemistry.

[36] Liu L., Gao Y., Geng W., Song J., Zhou Y., Li C. (2023). Comparison of jack bean and soybean crude ureases on surface stabilization of desert sand via enzyme-induced carbonate precipitation. Geoderma.

[37] Dilrukshi R.A.N., Nakashima K., Kawasaki S. (2018). Soil improvement using plant-derived urease-induced calcium carbonate precipitation. Soils Found..

[38] Yan B., Zhou Y., Li C., Shu S., Gao Y. (2023). Modified SICP method to mitigate the effect of bio-clogging by excess protein from soybean crude urease extracts for biocementation process. Acta Geotech..

[39] Miao L., Wu L., Sun X., Li X., Zhang J. (2020). Method for solidifying desert sands with enzyme-catalysed mineralization. Land Degrad. Dev..

[40] Ahenkorah I., Rahman M.M., Karim M.R., Beecham S. (2021). Enzyme induced calcium carbonate precipitation and its engineering application: A systematic review and meta-analysis. Constr. Build. Mater..

[41] Cui M.-J., Lai H.-J., Hoang T., Chu J. (2021). One-phase-low-pH enzyme induced carbonate precipitation (EICP) method for soil improvement. Acta Geotech..

[42] Hoang T., Alleman J., Cetin B., Ikuma K., Choi S.-G. (2019). Sand and silty-sand soil stabilization using bacterial enzyme-induced calcite precipitation (BEICP). Can. Geotech. J..

[43] Yi H., Zheng T., Jia Z., Su T., Wang C. (2021). Study on the influencing factors and mechanism of calcium carbonate precipitation induced by urease bacteria. J. Cryst. Growth.

[44] Zhang J.X., Xiao Y., Cui H., He X., Liu H.L. (2024). Dancing with crystals: Bacterial functions and interactions in biomineralization. Biogeotechnics.

[45] De Philippis R., Colica G., Micheletti E. (2011). Exopolysaccharide-producing cyanobacteria in heavy metal removal from water: Molecular basis and practical applicability of the biosorption process. Appl. Microbiol. Biotechnol..

[46] Nwodo U.U., Green E., Okoh A.I. (2012). Bacterial Exopolysaccharides: Functionality and Prospects. Int. J. Mol. Sci..

[47] Jittawuttipoka T., Planchon M., Spalla O., Benzerara K., Guyot F., Cassier-Chauvat C., Chauvat F. (2013). Multidisciplinary Evidences that *Synechocystis* PCC6803 Exopolysaccharides Operate in Cell Sedimentation and Protection against Salt and Metal Stresses. PLoS ONE.

[48] Kim H.J., Shin B., Lee Y.S., Park W. (2017). Modulation of calcium carbonate precipitation by exopolysaccharide in *Bacillus* sp JH7. Appl. Microbiol. Biotechnol..

[49] Shao P.P., Comolli L.R., Bernier-Latmani R. (2014). Membrane Vesicles as a Novel Strategy for Shedding Encrusted Cell Surfaces. Minerals.

[50] Zhu T., Dittrich M. (2016). Carbonate Precipitation through Microbial Activities in Natural environment, and Their Potential in Biotechnology: A Review. Front. Bioeng. Biotechnol..

[51] Oggerin M., Tornos F., Rodriguez N., del Moral C., Sanchez-Roman M., Amils R. (2013). Specific jarosite biomineralization by *Purpureocillium lilacinum*, an acidophilic fungi isolated from Rio Tinto. Environ. Microbiol..

[52] Gomez M.G., Anderson C.M., Graddy C.M.R., DeJong J.T., Nelson D.C., Ginn T.R. (2017). Large-Scale Comparison of Bioaugmentation and Biostimulation Approaches for Biocementation of Sands. J. Geotech. Geoenviron. Eng..

[53] Graddy C.M.R., Gomez M.G., DeJong J.T., Nelson D.C. (2021). Native Bacterial Community Convergence in Augmented and Stimulated Ureolytic MICP Biocementation. Environ. Sci. Technol..

[54] Gomez M.G., DeJong J.T., Anderson C.M. (2018). Effect of bio-cementation on geophysical and cone penetration measurements in sands. Can. Geotech. J..

[55] Cheng L., Shahin M.A., Cord-Ruwisch R. (2017). Surface Percolation for Soil Improvement by Biocementation Utilizing *In Situ* Enriched Indigenous Aerobic and Anaerobic Ureolytic Soil Microorganisms. Geomicrobiol. J..

[56] Wang X.R., Li C., He J. (2022). A highly effective strain screened from soil and applied in cementing fine sand based on MICP-bonding technology. J. Biotechnol..

[57] Harkes M.P., van Paassen L.A., Booster J.L., Whiffin V.S., van Loosdrecht M.C.M. (2010). Fixation and distribution of bacterial activity in sand to induce carbonate precipitation for ground reinforcement. Ecol. Eng..

[58] Ramachandran S.K., Ramakrishnan V., Bang S.S. (2001). Remediation of concrete using micro-organisms. ACI Mater. J..

[59] Whiffin V.S., van Paassen L.A., Harkes M.P. (2007). Microbial carbonate precipitation as a soil improvement technique. Geomicrobiol. J..

[60] Wei R., Peng J., Li L., Jiang Z., Tang J. (2023). Accelerated Reinforcement of Calcareous sand via Biomineralization with Aluminum Ion Flocculant. Appl. Biochem. Biotechnol..

[61] Cheng L., Shahin M.A., Chu J. (2019). Soil bio-cementation using a new one-phase low-pH injection method. Acta Geotech..

[62] Berg H. (1999). Problems of weak electromagnetic field effects in cell biology. Bioelectrochem. Bioenerg..

[63] Gao F., Feng J., Yan Z., Zhang M., Qin J., Zhang Y., Yang M. (2024). Weak electric field strengthens the β-oxidation degradation of fatty acids by activated sludge to produce micro-nano CaCO_3_. J. Environ. Chem. Eng..

[64] Zhu X., Lin F., Sun J., Li X., Zhu G., Lu Y., Sun L., Wang H. (2024). Effects of Weak Electric Fields on the Denitrification Performance of *Pseudomonas stutzeri*: Insights into Enzymes and Metabolic Pathways. Microorganisms.

[65] Deng J., Li M., Tian Y., Zhang Z., Wu L., Hu L. (2023). Using Electric Field to Improve the Effect of Microbial-Induced Carbonate Precipitation. Sustainability.

[66] Shao G., Huang R., Liu P. (2024). Application of electric fields to improve cementation homogeneity of biogrouted sands. Soil Use Manag..

[67] Deng J., Li M., Tian Y., Wu L., Hu L., Zhang Z., Zheng H. (2023). Experimental study on solidification of uranium tailings by microbial grouting combined with electroosmosis. Nucl. Eng. Technol..

[68] Achal V., Mukherjee A., Basu P.C., Reddy M.S. (2009). Strain improvement of *Sporosarcina pasteurii* for enhanced urease and calcite production. J. Ind. Microbiol. Biotechnol..

[69] Xu K., Huang M., Zhang J., Cui M., Xu C. (2023). Bio-cementation of tailings sands using ultraviolet-induced urease-producing bacteria. Environ. Geotech..

[70] Zhang J., Zhao C., Yin Y., Shi L., Bian H., Han Z. (2023). Experimental study on solidification of silt through urease-producing strains induced by ultraviolet mutagenesis. Chin. J. Geotech. Eng..

[71] Hawrylik E. (2019). The usage of ultrasounds to disintegrate escherichia Coli becteria contained in treated wastewater. Archit. Civ. Eng. Environ..

[72] Liu Z., Peng J., Li J., Song E. (2020). Experimental study on improving effect of microorganism solidifying sand by ultrasonic. J. Zhejiang Univ. Eng. Sci..

[73] Haystead J., Gilmour K., Sherry A., Dade-Robertson M., Zhang M. (2024). Effect of (in)organic additives on microbially induced calcium carbonate precipitation. J. Appl. Microbiol..

[74] Benini S., Rypniewski W.R., Wilson K.S., Miletti S., Ciurli S., Mangani S. (1999). A new proposal for urease mechanism based on the crystal structures of the native and inhibited enzyme from *Bacillus pasteurii*: Why urea hydrolysis casts two nickels. Structure.

[75] Won H.S., Lee Y.H., Kim J.H., Shin I.S., Lee M.H., Lee B.J. (2004). Structural characterization of the nickel-binding properties of *Bacillus pasteurii* urease accessory protein (Ure)E in solution. J. Biol. Chem..

[76] Carlsson H., Nordlander E. (2010). Computational Modeling of the Mechanism of Urease. Bioinorg. Chem. Appl..

[77] Dikshit R., Gupta N., Dey A., Viswanathan K., Kumar A. (2022). Microbial induced calcite precipitation can consolidate martian and lunar regolith simulants. PLoS ONE.

[78] Sun X., Miao L., Tong T., Wang C. (2018). Comparison between microbiologically-induced calcium carbonate precipitation and magnesium carbonate precipitation. Chin. J. Geotech. Eng..

[79] Lv C., Tang C.S., Zhang J.Z., Pan X.H., Liu H. (2023). Effects of calcium sources and magnesium ions on the mechanical behavior of MICP-treated calcareous sand: Experimental evidence and precipitated crystal insights. Acta Geotech..

[80] Wei R., Peng J., Chen Y., Xu P., Li L. (2023). Study on Methods and Effects of Coral Sand Reinforcement by MICP Combined with Reef Resources in South China Sea. J. Disaster Prev. Mitig. Eng..

[81] Li M., Cheng X., Yang Z., Guo H. (2013). Breeding High-yield Urease-producing *Sporosarcina pasteurii* Strain by NTG Mutation. J. Agric. Sci. Technol..

[82] Xiao Y., Deng H., Li J., Xiong Y., Cheng L. (2024). Effect of Triton X-100 on Improving Urease Activity of *Sporosarcina pasteurii*. Mater. Rev..

[83] Xu W., Zheng J., Chu J., Zhang R., Cui M., Lai H., Zeng C. (2021). New method for using N-(N-butyl)-thiophosphoric triamide to improve the effect of microbial induced carbonate precipitation. Constr. Build. Mater..

[84] Xu W., Zheng J., Cui M., Lai H. (2022). Study on the effect of MICP with NBPT on calcium carbonate deposition. J. Huazhong Univ. Sci. Technol. Nat. Sci..

[85] Gu Z.R., Li X.J., Wu J., Niu S., Wang P.B., Zheng J.J., Yan J.Y., Xu L., Yang M., Yan Y.J. (2023). A novel strategy for reinforcing cementation process coupling microbially induced carbonate precipitation (MICP) with cross-linked silk fibroin. J. Environ. Chem. Eng..

[86] Xue J.L., Wu Y.N., Shi K., Xiao X.F., Gao Y., Li L., Qiao Y.L. (2019). Study on the degradation performance and kinetics of immobilized cells in straw-alginate beads in marine environment. Bioresour. Technol..

[87] Kang C.-H., Kwon Y.-J., So J.-S. (2016). Bioremediation of heavy metals by using bacterial mixtures. Ecol. Eng..

[88] Zhu S., Hu X., Zhao Y., Fan Y., Wu M., Cheng W., Wang P., Wang S. (2020). Coal Dust Consolidation Using Calcium Carbonate Precipitation Induced by Treatment with Mixed Cultures of Urease-Producing Bacteria. Water Air Soil Pollut..

[89] Gat D., Tsesarsky M., Shamir D., Ronen Z. (2014). Accelerated microbial-induced CaCO_3_ precipitation in a defined coculture of ureolytic and non-ureolytic bacteria. Biogeosciences.

[90] Harnpicharnchai P., Mayteeworakoon S., Kitikhun S., Chunhametha S., Likhitrattanapisal S., Eurwilaichitr L., Ingsriswang S. (2022). High level of calcium carbonate precipitation achieved by mixed culture containing ureolytic and nonureolytic bacterial strains. Lett. Appl. Microbiol..

[91] Zhang J., Zhao C., Zhou A., Yang C., Zhao L., Li Z. (2019). Aragonite formation induced by open cultures of microbial consortia to heal cracks in concrete: Insights into healing mechanisms and crystal polymorphs. Constr. Build. Mater..

[92] Liu Y., Feng J., Pan H., Zhang X., Zhang Y. (2022). Genetically engineered bacterium: Principles, practices, and prospects. Front. Microbiol..

[93] Connolly J., Kaufman M., Rothman A., Gupta R., Redden G., Schuster M., Colwell F., Gerlach R. (2013). Construction of two ureolytic model organisms for the study of microbially induced calcium carbonate precipitation. J. Microbiol. Methods.

[94] Bergdale T.E., Pinkelman R.J., Hughes S.R., Zambelli B., Ciurli S., Bang S.S. (2012). Engineered biosealant strains producing inorganic and organic biopolymers. J. Biotechnol..

[95] Li F., Li X., Ye L., Liu X., Zhu J., Yang S., Yan Y., Xu L., Yan J. (2022). A genetically engineered composite biofilm for microbial induced calcium carbonate precipitation by synergic effect of urease, protein adhesive and xanthan gum. J. Environ. Chem. Eng..

[96] Gorospe C.M., Han S.-H., Kim S.-G., Park J.-Y., Kang C.-H., Jeong J.-H., So J.-S. (2013). Effects of different calcium salts on calcium carbonate crystal formation by *Sporosarcina pasteurii* KCTC 3558. Biotechnol. Bioprocess Eng..

[97] Lauchnor E.G., Topp D.M., Parker A.E., Gerlach R. (2015). Whole cell kinetics of ureolysis by *Sporosarcina Pasteurii*. J. Appl. Microbiol..

[98] Ng W.S., Lee M., Hii S.L. (2012). An Overview of the Factors Affecting Microbial-Induced Calcite Precipitation and its Potential Application in Soil Improvement. J. Civ. Environ. Eng..

[99] Omoregie A.I., Khoshdelnezamiha G., Senian N., Ong D.E.L., Nissom P.M. (2017). Experimental optimisation of various cultural conditions on urease activity for isolated *Sporosarcina pasteurii* strains and evaluation of their biocement potentials. Ecol. Eng..

[100] Dong Y., Gao Z., Wang D., Di J., Guo X., Yang Z., Li Y., Wang Y., Wang Y. (2023). Optimization of growth conditions and biological cementation effect of *Sporosarcina pasteurii*. Constr. Build. Mater..

[101] Han Q., Xiao Y., Li P. (2023). Study on the effects of different polymicrobial environments on b. Pasteurii survivability, urease activity, and crack healing. Constr. Build. Mater..

[102] Shi W.B., Wang M., Wu L., Xie X.H., Wang M., Lu T.J. (2022). Study of Concrete Crack Repair using *Bacillus megaterium*. Adv. Mater. Sci. Eng..

[103] Sun X., Miao L., Tong T., Wang C. (2018). Effect of methods of adding urea in culture media on sand solidification tests. Chin. J. Geotech. Eng..

[104] Sun X., Miao L., Wu L., Wang C., Chen R. (2019). Experimental study on precipitation rate of MICP under low temperatures. Chin. J. Geotech. Eng..

[105] Peng J., Cao T., He J., Dai D., Tian Y. (2022). Improvement of Coral Sand with MICP Using Various Calcium Sources in Sea Water Environment. Front. Phys..

[106] Xiao Y., Deng H., Li J., Cheng L., Zhu W. (2022). Study on the domestication of *Sporosarcina pasteurii* and strengthening effect of calcareous sand in seawater environment. Rock Soil Mech..

[107] Guo S.J., Zhang J.X., Li M., Zhou N., Song W.J., Wang Z.J., Qi S.M. (2021). A preliminary study of solid-waste coal gangue based biomineralization as eco-friendly underground backfill material: Material preparation and macro-micro analyses. Sci. Total Environ..

[108] Farmani F., Bonakdarpour B., Ramezanianpour A.A. (2015). pH reduction through amendment of cement mortar with silica fume enhances its biological treatment using bacterial carbonate precipitation. Mater. Struct..

[109] Su Y., Zheng T., Qian C. (2021). Application potential of *Bacillus megaterium* encapsulated by low alkaline sulphoaluminate cement in self-healing concrete. Constr. Build. Mater..

[110] Antipov A., Shchukin D., Fedutik Y., Zanaveskina I., Klechkovskaya V., Sukhorukov G., Möhwald H. (2003). Urease-catalyzed carbonate precipitation inside the restricted volume of polyelectrolyte capsules. Macromol. Rapid Commun..

[111] Seifan M., Sarmah A.K., Ebrahiminezhad A., Ghasemi Y., Samani A.K., Berenjian A. (2018). Bio-reinforced self-healing concrete using magnetic iron oxide nanoparticles. Appl. Microbiol. Biotechnol..

[112] Wang J.Y., De Belie N., Verstraete W. (2012). Diatomaceous earth as a protective vehicle for bacteria applied for self-healing concrete. J. Ind. Microbiol. Biotechnol..

[113] Jiang L., Jia G., Jiang C., Li Z. (2020). Sugar-coated expanded perlite as a bacterial carrier for crack-healing concrete applications. Constr. Build. Mater..

[114] Wang J.Y., Snoeck D., Van Vlierberghe S., Verstraete W., De Belie N. (2014). Application of hydrogel encapsulated carbonate precipitating bacteria for approaching a realistic self-healing in concrete. Constr. Build. Mater..

[115] Soysal A., Milla J., King G.M., Hassan M., Rupnow T. (2020). Evaluating the Self-Healing Efficiency of Hydrogel-Encapsulated Bacteria in Concrete. Transp. Res. Rec..

[116] Cui M.-J., Zheng J.-J., Zhang R.-J., Lai H.-J. (2020). Soil bio-cementation using an improved 2-step injection method. Arab. J. Geosci..

[117] Yang Y., Chu J., Liu H., Cheng L. (2023). Improvement of uniformity of biocemented sand column using CH_3_COOH-buffered one-phase-low-pH injection method. Acta Geotech..

[118] Yu X., Yang H. (2023). One-phase MICP and two-phase MISP composite cementation. Constr. Build. Mater..

[119] Lai Y., Liu S., Cai Y., Yu J. (2023). Reinforcement of Different Sands by Low-pH Bio-Mineralization. Materials.

[120] Cui M.-J., Lai H.-J., Hoang T., Chu J. (2022). Modified one-phase-low-pH method for bacteria or enzyme-induced carbonate precipitation for soil improvement. Acta Geotech..

[121] Lai Y., Yu J., Liu S., Liu J., Wang R., Dong B. (2021). Experimental study to improve the mechanical properties of iron tailings sand by using MICP at low pH. Constr. Build. Mater..

[122] Lai H.-J., Cui M.-J., Chu J. (2023). Effect of pH on soil improvement using one-phase-low-pH MICP or EICP biocementation method. Acta Geotech..

[123] Zhang J., Liu W., Guan D., Zhou Y., Cheng L., Zheng J. (2020). Influence of different bacterial grouting strategies on MICP one-phase injection method. J. Hohai Univ. Nat. Sci..

[124] Li L., Liu T., Jiang G., Fang C., Qu B., Zheng S., Yang G., Tang C. (2022). Insight into the temperature stimulation on the self-healing properties of cement-based materials. Constr. Build. Mater..

[125] Kalantary F., Kahani M. (2019). Optimization of the biological soil improvement procedure. Int. J. Environ. Sci. Technol..

[126] Xiao Y., Wang Y., Wang S., Evans T.M., Stuedlein A.W., Chu J., Zhao C., Wu H., Liu H. (2021). Homogeneity and mechanical behaviors of sands improved by a temperature-controlled one-phase MICP method. Acta Geotech..

[127] Xiao P., Liu H., Stuedlein A.W., Evans T.M., Xiao Y. (2019). Effect of relative density and biocementation on cyclic response of calcareous sand. Can. Geotech. J..

[128] Xiao Y., Wang Y., Desai C.S., Jiang X., Liu H. (2019). Strength and Deformation Responses of Biocemented Sands Using a Temperature-Controlled Method. Int. J. Geomech..

[129] Lv C., Tang C.-S., Zhang J.-Z., Liu H., Pan X.-H. (2024). Regulating the Process of Microbially Induced Calcium Carbonate Precipitation through Applied Electric Fields: Evidence and Insights Using Microfluidics. J. Geotech. Geoenviron. Eng..

[130] Wang Y., Soga K., Dejong J.T., Kabla A. (2019). A microfluidic chip and its use in characterising the particle-scale behaviour of microbial-induced calcium carbonate precipitation (MICP). Geotechnique.

[131] Xiao Y., He X., Stuedlein A.W., Chu J., Evans T.M., van Paassen L.A. (2022). Crystal Growth of MICP through Microfluidic Chip Tests. J. Geotech. Geoenviron. Eng..

[132] Su F., Yang Y., Qi Y., Zhang H. (2022). Combining microbially induced calcite precipitation (MICP) with zeolite: A new technique to reduce ammonia emission and enhance soil treatment ability of MICP technology. J. Environ. Chem. Eng..

[133] Yuan H., Zhang Q., Hu X., Wu M., Zhao Y., Feng Y., Shen D. (2022). Application of zeolite as a bacterial carrier in the self-healing of cement mortar cracks. Constr. Build. Mater..

[134] Lajevardi S.H., Shafiei H. (2023). Investigating the biological treatment effect on fine-grained soil resistance against wind erosion: An experimental case study. Aeolian Res..

[135] Meng H., Gao Y., He J., Qi Y., Hang L. (2021). Microbially induced carbonate precipitation for wind erosion control of desert soil: Field-scale tests. Geoderma.

